# An adaptive threshold determination method of feature screening for genomic selection

**DOI:** 10.1186/s12859-017-1617-9

**Published:** 2017-04-12

**Authors:** Guifang Fu, Gang Wang, Xiaotian Dai

**Affiliations:** Department of Mathematics and Statistics, Utah State University, Logan, 84322 UT USA

**Keywords:** Genomic selection, Feature screening, Backward elimination, *FRIGIDA* expression

## Abstract

**Background:**

Although the dimension of the entire genome can be extremely large, only a parsimonious set of influential SNPs are correlated with a particular complex trait and are important to the prediction of the trait. Efficiently and accurately selecting these influential SNPs from millions of candidates is in high demand, but poses challenges. We propose a backward elimination iterative distance correlation (BE-IDC) procedure to select the smallest subset of SNPs that guarantees sufficient prediction accuracy, while also solving the unclear threshold issue for traditional feature screening approaches.

**Results:**

Verified through six simulations, the adaptive threshold estimated by the BE-IDC performed uniformly better than fixed threshold methods that have been used in the current literature. We also applied BE-IDC to an *Arabidopsis thaliana* genome-wide data. Out of 216,130 SNPs, BE-IDC selected four influential SNPs, and confirmed the same *FRIGIDA* gene that was reported by two other traditional methods.

**Conclusions:**

BE-IDC accommodates both the prediction accuracy and the computational speed that are highly demanded in the genomic selection.

**Electronic supplementary material:**

The online version of this article (doi:10.1186/s12859-017-1617-9) contains supplementary material, which is available to authorized users.

## Background

Genomic selection is an important task for increasing the efficiency of plant breeding, disease diagnosis, personalized medicine, and genotyping chip design. Genomic selection is improved by identifying a small subset of influential single nucleotide polymorphisms (SNPs) from high-dimensional genetic information to efficiently predict individual’s phenotype [1–5]. The rapid developments of high-throughput genomic technologies, such as whole genome genotyping, next generation sequencing, gene expression microarray, and RNA-seq, have dramatically boosted the landscape and power of genomic selection [6, 7], while nevertheless bringing unprecedented challenges for statistical modeling.

Feature screening has been receiving extensive attention as a powerful approach to handle ultrahigh dimensional data, which is defined as *p*= exp(*n* ^*ζ*^), for some *ζ*>0. Here *p* is the number of features and *n* is the number of observations [8–19]. Specifically, Li et al. developed a distance correlation based sure independence feature screening (DC-SIS) strategy that defines an association strength measure for each feature based on its distance correlation with the phenotype [16]. The idea of DC-SIS is to theoretically satisfies the sure screening property, ranks the features from the most important to the least important by decreasing distance correlation values, and filters the majority of noise with low values of the defined association strength measure. A very attractive property of DC-SIS is that it effectively captures both the linear and nonlinear association between the feature and the phenotype, and feasible for binary, continuous, and categorical features and phenotype, without assuming any specific model structure, distribution, or data type. In addition, DC-SIS outperforms the traditional sure independence screening (SIS) [9] and sure independent ranking and screening (SIRS) approaches [13]. Therefore, DC-SIS has great potential in serving genomic selection and recognizing the truly influential SNPs from millions of candidates covering the entire genome.

However, a limitation that restricts the application of DC-SIS and other feature screening approaches is the lack of a clear threshold determination to separate influential features from noise, which is crucial in genomic selection. Frequently, an arbitrary decision as to the number of genes to retain is made. For example, some papers used sheer empirical experience to select the 50 or 150 highest ranked genes [20–22]. Other traditional approaches selected the SNPs passing the threshold of −*log*(0.05/*p*) [23–26]. However, this approach requires that a p-value is accessible, and hence annuls the possibility of applying any approaches that do not compute a p-value. Current feature screening literature keeps the top *d* features having the highest rankings, where *d* is often computed from an integral multiplier of [n/log(n)] (e.g., *d*_1_=[ *n*/*log*(*n*)],*d*_2_=2[ *n*/*log*(*n*)], or *d*_3_=3[ *n*/*log*(*n*)] is suggested) [9, 16, 17, 27]. These options may work well for some circumstances, but several drawbacks also present themselves: 1) It is still not clear what *d* exactly should be. For a real data analysis, we have no any idea whether *d*_1_, *d*_2_, *d*_3_, or an even larger value should be used. 2) A formula such as [ *n*/*log*(*n*)] is only restricted by the sample size, but unreasonably neglect two indispensable considerations: the number of features, and the signal-to-noise ratio. Assuming the sample size is fixed, it is unreasonable to set the same threshold for one dataset with 100 features and another dataset with 100,000 features. For a dataset with a large number of features, but a weak signal-to-noise ratio, the threshold may be relatively large, whereas for a much easier scenario, the threshold may be small. Kong et al. recognized these limitations and proposed a theorem to adaptively determine the threshold for DC-SIS, pioneering threshold determination research [28]. However, they assumed that noise SNP was purely independent of the influential SNPs and the phenotype, which may not be true in the genome-wide datasets.

In particular, determining a threshold that separates influential SNPs from noise SNPs is a necessity in genomic selection. But method for determining the threshold is also limited in the genomic selection literature. Frequently, an arbitrary decision as to the number of genes to retain was made. For example, some papers used sheer empirical experience to select the 50 or 150 highest ranked genes. Other traditional approaches selected the SNPs passing the threshold of −*log*(0.05/*p*). However, this approach requires that a p-value is accessible, and hence annuls the possibility of applying many other feature screening approaches which do not compute a *p*-value.

This article extends the work of Zhong et al. [27], and proposes a backward elimination iterative distance correlation (BE-IDC) procedure that adaptively and automatically determines an optimal threshold for genomic selection while guaranteeing prediction accuracy. The smoothly clipped absolute deviation (SCAD) penalized regression model fitting the bootstrap samples is used to compute the mean square prediction error (MSPE). A certain percentage of SNPs (controlled by the drop rate) are backward eliminated after iterative DC-SIS ranks the SNPs from the most important to the least important, and the point at which the minimum MSPE is attained is determined as the final threshold. One aim is to optimize the threshold, which is not trivial. If the standard is too stringent and the number of SNPs selected is too small, we may fail to cover all influential SNPs, and hence the power will be reduced; whereas if the rule is too liberal and the number of SNPs selected is too large, too much of the noise will be mistreated as influential features, and hence the false discovery rate will be inflated [29]. Another aim is to obtain the smallest possible set of SNPs that can still achieve acceptable prediction accuracy. The proposed BE-IDC realizes these two aims, serves as a good genomic selection procedure for the ultrahigh dimensional genome-wide dataset, and overcomes the limitation of previous feature screening approaches.and boosts the potential of feature screening approaches to bring a new horizon for genomic selection

We explored the performances of the BE-IDC approach using six different simulation settings and one real genome-wide dataset. We also compared the results of the adaptive threshold estimated by BE-IDC with those found by the fixed thresholds suggested by current feature screening literature [9, 16, 17, 27]. The average thresholds estimated by BE-IDC are uniformly lower than the fixed thresholds while yet achieving 100% power. It is worth mention that the BE-IDC uses an average threshold as small as 5.54 to achieve a 100% power for Example 1, which is only 7% of the fixed threshold, *d*=2[ *n*/*log*(*n*)]=74, used by the current literature [9, 27]. In addition, BE-IDC can flexibly and automatically adjust the threshold when the dataset has harder conditions (see Example 3 and Additional file 1: Supplementary example 1). From these six simulations, we conclude that BE-IDC shows uniformly excellent performances even when the signal-to-noise ratio is low (e.g., only four influential features are truly associated with the phenotype, and 4996 features are noise), and when the number of features is much larger than the number of observations (e.g., *p*=5,000 and *n*=200). We also demonstrate that the BE-IDC approach selects a very small set of SNPs for *Arabidopsis thaliana* data. Here, only four SNPs are selected from a pool of 216,130 SNPs covering the entire genome, and the *FRIGIDA* gene, reported to be highly associated with the *FRIGIDA* expression trait being analyzed [23], is successfully picked out.

## Methods

### Iterative DC-SIS

Szekely et al. defined an association strength measure for each feature based on its distance correlation (Dcorr) with the phenotype and showed that the Dcorr of two random vectors equals zero if and only if the two random vectors are independent [30]. Li et al. proposed the DC-SIS feature screening approach, ranked the SNPs from the most important to the least important by decreasing the values of Dcorr, and proved the sure screening theorem to theoretically ensure that DC-SIS will not miss any influential SNPs if the sample size is large enough [16].

Let **y** be the analyzed phenotype. Let **X**_*j*_ be the genotype of each SNP, *j*=1,…,*p*. For each biallelic locus, the three possible genotypes can be coded as 0 (for aa), 1 (for Aa), and 2 (for AA). The distance covariance between the phenotype and each SNP is defined as

1 $$ \begin{aligned} dcov^{2}(\mathbf{y},\mathbf{X}_{j})&=\int \left\|\phi_{\mathbf{y},\mathbf{X}_{j}}(t,s)-\phi_{\mathbf{y}}(t)\phi_{\mathbf{X}_{j}}(s)\right\|^{2}\\ &\quad \times w(t,s)dtds, \end{aligned}  $$

where *ϕ*_**y**_(*t*) and $\phantom {\dot {i}\!}\phi _{\mathbf {X}_{j}}(s)$ are the respective characteristic functions of **y** and **X**_*j*_, and $\phantom {\dot {i}\!}\phi _{\mathbf {y},\mathbf {X}_{j}}(t,s)$ is the joint characteristic function of (**y**,**X**_*j*_), and

$$w(t,s)=\left\{\pi^{2}~\|t\|^{2} ~\|s\|^{2}\right\}^{-1}, $$

where ||·|| stands for the Euclidean norm. Then the Dcorr between the phenotype and each SNP is defined as

2 $$ Dcorr\left(\mathbf{y},\mathbf{X}_{j}\right)=\frac{dcov\left(\mathbf{y},\mathbf{X}_{j}\right)} {\sqrt{dcov\left(\mathbf{y},\mathbf{y}\right)~dcov\left(\mathbf{X}_{j},\mathbf{X}_{j}\right)}}.  $$

Szekely et al. gave a numerically easier estimator of $\widehat {dcov}^{2}(\mathbf {y},\mathbf {X}_{j})$ as

3 $$ \widehat{dcov}^{2}\left(\mathbf{y},\mathbf{X}_{j}\right)=\hat{S}_{1}+\hat{S}_{2}-2\hat{S}_{3}.  $$

Let **y**_*i*1_, **y**_*i*2_, **X**_*i*1,*j*_, and **X**_*i*2,*j*_ denote the $i_{1}^{th}$ and $i_{2}^{th}$ sample observations for **y** and **X**_*j*_, respectively. Then

4 $$ { \begin{aligned} \hat{S}_{1} &= \frac{1}{n^{2}} \sum_{i1=1}^{n} \sum_{i2=1}^{n} \left\|\mathbf{y}_{i1}-\mathbf{y}_{i2}\right\|~\left\|\mathbf{X}_{i1,j}-\mathbf{X}_{i2,j} \right\|_{p}\\ \hat{S}_{2} &= \frac{1}{n^{2}} \sum_{i1=1}^{n} \sum_{i2=1}^{n} \left\|\mathbf{y}_{i1}-\mathbf{y}_{i2}\right\|~ \frac{1}{n^{2}} \sum_{i1=1}^{n} \sum_{i2=1}^{n} \left\|\mathbf{X}_{i1,j}-\mathbf{X}_{i2,j} \right\|_{p}, \\ \hat{S}_{3} &= \frac{1}{n^{3}} \sum_{i1=1}^{n} \sum_{i2=1}^{n} \sum_{i3=1}^{n} \left\|\mathbf{y}_{i1}-\mathbf{y}_{i3}\right\|~\left\|\mathbf{X}_{i2,j}-\mathbf{X}_{i3,j} \right\|_{p}. \end{aligned}}  $$

Finally, the point estimator $\widehat {Dcorr}(y,\mathbf {X}_{j})$ can be estimated by Eqs. (2), (3) and (4). We are then able to rank all SNPs, from the most influential to the least influential, by decreasing values of $\widehat {Dcorr}(y,\mathbf {X}_{j}), j=1,\ldots,p$ [16]. Let $\mathbf {X}_{p}=\{X^{*}_{k}, k=1,\ldots,p\},$ be the reordered SNPs, where the asterisk is used to differentiate the top *k* ^*th*^ SNP after selection by DC-SIS from the originally observed *k* ^*th*^ SNP.

While DC-SIS is a very powerful feature selection approach for ultrahigh dimensional data, it may neglect some important SNPs that are marginally not relevant, but jointly associated with the phenotype; or, it may rank highly some noise SNPs that are spuriously correlated with the phenotype due to their strong linkage disequilibrium (LD) with other important SNPs. To overcome these shortcomings, Zhong et al. introduced an iterative distance correlation feature screening approach (IDC-SIS) [27]. The main idea of IDC-SIS is to iteratively regress unselected SNPs on selected SNPs, regain information from the residuals, and effectively break down the effects of correlation structure among SNPs.

DC-SIS ranks all SNPs and achieves the set **X**_*p*_ in a single step, while IDC-SIS builds up **X**_*p*_ gradually with several steps, i.e. $\mathbf {X}_{p}=\mathbf {X}_{p1} \bigcup \mathbf {X}_{p2} \bigcup \ldots \bigcup \mathbf {X}_{pm}$, with *p*=*p*_1_+*p*_2_+…+*p*_*m*_, where **X**_*pi*_ stands for the set of SNPs selected at the *i* ^*th*^ iterative step, *p*_*i*_ denotes the size of each set **X**_*pi*_; *i*=1,…,*m*, and *m* is the number of iterative steps. Zhong et al. claimed that a small number of iterations is adequate to guarantee good performance and they suggested *m*=2,*p*_1_=5,*p*_2_=*d*−5, and *d*=2[*n*/*logn*] [27]. Note that this article aims to adaptively determine *d* without assuming it is given, hence we set *m*=3,*p*_1_=3,*p*_2_=3, and *p*_3_=*p*−6 to rank all features. This setting is empirically proven to work well in all simulations.

The details of IDC-SIS can be summarized as follows [27]:

- Step 1: Use DC-SIS for **y** and **X** and select the first *p*_1_ features into **X**_*p*_ (i.e. **X**_*p*_=**X**_*p*1_).
- Step 2: Define $\mathbf {X}_{r}=\{I_{n}-\mathbf {X}_{p}(\mathbf {X}_{p}^{T}\mathbf {X}_{p})^{-1}\mathbf {X}_{p}^{T}\}\mathbf {X}_{p}^{C}$, where $\mathbf {X}_{p}^{C}$ is the complement set of **X**_*p*_. Use DC-SIS for **y** and **X**_*r*_ and select the second *p*_2_ features into **X**_*p*_ (i.e. $\mathbf {X}_{p}=\mathbf {X}_{p1} \bigcup \mathbf {X}_{p2}$).
- Step 3: Repeat Step 2 for *m* times until all *p* features are ranked, i.e., $\mathbf {X}_{p}=\mathbf {X}_{p1} \bigcup \mathbf {X}_{p2} \bigcup \ldots \bigcup \mathbf {X}_{pm}$, with *p*=*p*_1_+*p*_2_+…+*p*_*m*_. Note that the computational cost will be shockingly large if we repeat step 2 for too many times for a large number of SNPs. Additionally, the theoretical sure screening property may not continue to be true if too many iterations are applied. To balance the computational cost and accuracy, we only selected the first one hundred SNPs by IDC-SIS and then applied DC-SIS for all remaining SNPs. This combination worked well after verifying by quite a few empirical studies (see simulation section).

### Backward elimination

Let *d* denote the threshold that we need to determine. Let $\mathbf {X}_{\mathcal {C}}=\{X_{k}^{*}, k=1,\ldots,d\}$ be the subset of influential SNPs, i.e., the conditional distribution function of **y** depends on **X** merely through $\mathbf {X}_{\mathcal {C}}$, and let $\mathbf {X}_{\mathcal {N}}=\{X^{*}_{k}, k=d+1,\ldots,p\}$ be the set of noise SNPs, i.e., the complement set of $\mathbf {X}_{\mathcal {C}}$. The goal of genomic selection is to remove $\mathbf {X}_{\mathcal {N}}$ and pick the subset $\mathbf {X}_{\mathcal {C}}$. DC-SIS is able to rank important features before noise, but a genomic selection process cannot be finalized if *d* is not determined. The current feature screening literature suggests using a fixed threshold of *d*= [ *n*/*logn*] ([·] is the nearest integer function) [16, 27]. But again, this has several limitations as discussed in the Introduction section.

Starting from the biggest pool, **X**_*p*_, which contained all reordered SNPs as ranked by IDC-SIS, we discarded the noise SNPs by a backward elimination process through several iterations. For each iteration, we computed mean square prediction error based on its current pool, threw away a certain drop rate of SNPs from the bottom of the rank (i.e., those having the smallest Dcorr values), then moved to the next iteration step. The backward elimination considered all SNPs at the initial stage of the modeling to attenuate possible modeling biases.

### SCAD penalized regression model

To predict the phenotypic values for the test data while accommodating the ultrahigh dimensionality of the genome-wide data (in particular for the first couple of iterations of the backward elimination process), we applied a penalized regression model with the non-concave SCAD penalty function [31]. Unlike the traditional regression model, the penalized least squares estimators were obtained by minimizing

$$\frac{1}{2} (y-X\beta)^{T}(y-X\beta)+n \sum_{j=1}^{p} p_{\lambda}(|\beta_{j}|), $$

where the SCAD penalty function was given as

$$p_{\lambda}(|\beta_{j}|)= \left\{\begin{array}{ll} \lambda |\beta_{j}|, & |\beta_{j}|\leq \lambda; \\ -\frac{|\beta_{j}|^{2}-2\alpha \lambda |\beta_{j}|+\lambda^{2}}{2(\alpha-1)}, & \lambda < |\beta_{j}| \leq \alpha \lambda; \\ (\alpha+1)\lambda^{2}/2, & |\beta_{j}| > \alpha \lambda. \end{array}\right. $$

Two unknown tuning parameters *λ* and *α* are contained in the penalty function. As suggested by Fan et al. [31], *α*=3.7 is a good choice for various problems, and *λ* is selected by cross validation. This penalty function corresponds to a quadratic spline function with knots at *λ* and *α* *λ*. Besides its capability of handling ultrahigh dimensional genome-wide data, the SCAD penalty function satisfied three properties that are important for genomic selection: It is singular at the origin to produce sparse solutions and shrink unimportant parameters to zero to reduce model complexity; the resulting estimator is continuous, which retains stability in model prediction; and it is bounded by a constant to produce nearly unbiased estimates for large coefficients to avoid unnecessary modeling bias [31].

### Scheme of BE-IDC

The details of BE-IDC are summarized as follows:

- Step 1: Rank all SNPs by IDC-SIS, and obtain the reordered set (i.e., **X**_*p*_), where *p* is expected to be ultrahigh.
- Step 2: Start from the biggest pool (size of *p*), **X**_*p*_, and compute the MSPE for the corresponding model.
- Step 3: Remove a certain drop rate of SNPs having the lowest Dcorr values, based on the ranks obtained from Step 1. Then compute the MSPE for the model corresponding to the current pool. For more details about the drop rate, please see the simulation section.
- Step 4: Repeat Step 3 until the smallest pool (size of 1 at minimum) is reached.
- Step 5: Draw a plot of the MSPE versus the number of SNPs and locate the model size for which the MSPE is minimized, as model size decreases from *p* to 1. Finally, the selected influential SNP set (i.e., $\mathbf {X}_{\mathcal {C}}$) and the adaptive threshold (i.e., $\hat {d}$) can be simultaneously determined from this optimal spot. The noise set $\mathbf {X}_{\mathcal {N}}$ is already thrown away during the iterations of Steps 3 and 4.

The computation of the MSPE mentioned in above steps 2 and 3 is done as follows: Draw 1000 bootstrap samples with replacement, divide each bootstrap sample into training data (the observations being drawn) and test data (the observations not being drawn, also called out of bag (OOB) observations), fit the SCAD penalized regression model using the training data, predict for the test data, then compute the mean square prediction errors for all the bootstrap samples.

Following this BE-IDC scheme, the prediction accuracy and reproducibility of results on new datasets should be guaranteed because the minimum mean square prediction error is used. However, if a very small threshold is preferred, we suggest using the smallest number of SNPs whose MSPE is within 1 standard error (1 s.e. rule) above the minimum MSPE. In this case, the number of SNPs may be smaller than $\hat {d}$, and the prediction error a little larger than the minimum MSPE value but the MSPE will still lie within an acceptable range. However, it is expected that the power may decrease and influential SNPs may be missed if this 1 s.e. rule is used. Therefore, unless a very small number of SNPs is preferred for reason of saving experimental cost in breeding or disease diagnosis applications, we suggest taking the threshold to be that for which the MSPE is minimized.

## Results

### Simulation studies

The performances of DC-SIS and IDC-SIS have been investigated by Li et al. [16] and Zhong et al. [27] using a fixed threshold. In this section we examined the performance of the adaptive threshold estimated from the new approach BE-IDC through six simulation studies. Additionally, we compared the performance of the adaptive threshold with the fixed threshold $\hat {d}=2[\!n/log(n)]=74$ used by Li et al. [16] and Zhong et al. [27]. To make the comparisons fair, the first two examples were imitated from the current literature [9, 27, 32]. In the third example, we increased the level of rigor. As with the first two simulations, we also used the same criteria that have been widely used to assess the power of a method in current feature screening literature [16, 17, 27]:

- **Average Threshold** $\bar {\hat {d}}$: the average value of the adaptive thresholds estimated from the 100 simulation replicates;
- **Strict Power** *P*_*a*_: the proportion of the 100 replicates for which all truly influential features are simultaneously selected by $\hat {d}$;
- **Individual Power** *P*_*j*_: the proportion of the 100 replicates for which each individual influential feature is selected by $\hat {d}$ respectively.

We simulated 100 replicate datasets consisting of *n*=200 observations and *p*=5,000 features for Example 1-2 and Additional file 1: Supplementary example 1-2. We simulated 100 replicate datasets consisting of *n*=200 observations and *p*=2,000 features for Example 3 and Additional file 1: Supplementary example 3.

### Example 1

Similar to Zhong et al. [9, 27], we simulated data for Example 1 from the model

5 $$ y = 5X_{1} + 5X_{2} + 5X_{3} - 15\sqrt{\rho}X_{4} + \epsilon,   $$

where the features were generated from a normal distribution with zero mean, unit variance, and covariance structure following AR(1) design with auto correlation parameter *ρ*=0.5. Model (5) actually sets the first four features as the influential ones, and all remaining 4,996 features as noise. The white noise was generated from the standard normal distribution and the continuous phenotype *y* was generated from Model (5) accordingly.

Table 1 summarizes the simulation results of average thresholds $\bar {\hat {d}}$, strict power *P*_*a*_, and individual power *P*_*j*_, *j*=1,…,4, achieved by fixed threshold *d*=2[*n*/*log*(*n*)] and adaptive threshold, respectively. From Table 1, we can see that BE-IDC achieved 100% strict power using only 5.54 features on average after searching 5,000 features for 100 replications, while the fixed threshold used a threshold of 74, which is 13 times larger than the result of BE-IDC to achieve the same power. Simulation further indicated that BE-IDC only made an average of 1.54 spurious signal, but that the fixed threshold made an average of 70 spurious signals for 100 simulations (akin to a type I error rate). Note that *X*_4_ was jointly important but marginally independent to the phenotype **y**, so it trapped the DC-SIS (*P*_4_=0*%*) but was successfully detected by BE-IDC using a very small model size (*P*_4_=100*%*). In addition, we assessed the average of 100 mean square prediction errors using the 5-fold cross validation for these three methods, respectively. The BE-IDC achieved the best prediction accuracy (see Table 1). Given the fact that IDC-SIS already beat four other feature screening approaches [27], to wit, LASSO [33], sure independence screening (SIS) [9], iterative sure independence screening (ISIS) [9], and DC-SIS [16], this improvement is agreeable. The strict power of BE-DC (i.e., backward elimination distance correlation without iteration) is zero even using 9 times more features because *X*_4_ is purposely designed to be trapped by noise. This example illustrates that the benefits of BE-IDC over BE-DC.

**Table 1:** Strict and individual statistical power for methods using fixed or adaptive thresholds for Example 1

Methods	Average $\hat {d}$	*P*_*a*_	*P*_1_	*P*_2_	*P*_3_	*P*_4_	Average MSPE
DC-SIS (*d*=74)	74	0%	100%	100%	100%	0%	6.91
IDC-SIS (*d*=74)	74	100%	100%	100%	100%	100%	1.82
BE-IDC	5.54	100%	100%	100%	100%	100%	1.04

We also tested the sensitivity of different drop rates (i.e., the percentage of the noise SNPs discarded at each iteration, mentioned in Step 3 of the BE-IDC scheme) using this simulation study and empirically verified that the results are quite stable when drop rate varies dramatically from 10 to 50% (see Table 2). The thresholds, $\hat {d}$, have only negligible differences according to the five different resolutions of the drop rates. Throughout this article, we used a drop rate of 50% to save the computational cost and found that such a big drop rate is able to achieve high powers and small thresholds. But if a high resolution is needed and computational cost is not a concern, a smaller drop rate, say 10%, is suggested.

**Table 2:** Strict power and average threshold for BE-IDC approach under different drop rates

Drop Rate	Average $\hat {d}$	*P*_*a*_
50%	5.54	100%
40%	5.45	100%
30%	5.34	100%
20%	5.39	100%
10%	5.33	100%

### Example 2

We simulated SNP data for Example 2 following the procedure of Li et al. [32]. Firstly, *u*_*ij*_ was generated from a standard normal distribution with correlation structure of *ρ*=cov(*u*_*ij*_,*u*_*ik*_)=0.1. To simulate SNPs with equal allele frequencies, we set

$$ \mathbf{X}_{ij} =\left\{ \begin{array}{ll} AA~ (\text{coded as 2}), & u_{ij} > c \\ Aa~ (\text{coded as 1}), & -c \leq u_{ij} \leq c \\ aa~ (\text{coded as 0}), & u_{ij} < -c, \\ \end{array}\right. $$

where c is the third quartile of a standard normal distribution. Secondly, the additive ($X_{ij}^{a}$) and dominant feature ($X_{ij}^{d}$) of each SNP were coded as follows,

$$ \begin{aligned} &X_{ij}^{a} =\left\{ \begin{array}{lll} 1, & \text{if} & X_{ij} = AA \\ 0, & \text{if} & X_{ij} = Aa \\ -1, & \text{if} & X_{ij} = aa, \\ \end{array}\right.\\ &X_{ij}^{d} =\left\{ \begin{array}{lll} 1, & \text{if} & X_{ij} = Aa \\ 0, & \text{if} & X_{ij} = AA \quad \text{or} \quad aa. \\ \end{array}\right. \end{aligned} $$

Thirdly, we let the set *j*∈{100,200,300,400,500} contains the indices of truly influential SNPs, and the additive ($\beta _{j}^{a}$) and dominant coefficients ($\beta _{j}^{d}$) of the influential SNPs are given by Table 3.

**Table 3:** Genetic effects of 5 assumed SNPs in Example 2

Position	Additive ($\beta _{j}^{a}$)	Dominant ($\beta _{j}^{d}$)
100	1.2	0.8
200	1.2	0.4
300	1.2	0.8
400	0.8	1.2
500	1.0	1.2

Finally, the phenotype **y** was generated following Li et al.’s design [32],

6 $$ \mathbf{y}_{i} = \sum_{j} \beta_{j}^{a} X_{ij}^{a} + \sum_{j} \beta_{j}^{d}X_{ij}^{d} + \epsilon,   $$

where *ε*∼*N*(0,1). Note that **X**_*ij*_ is the feature that we analyzed, therefore the Example 2 connected **y** and **X**_*ij*_ indirectly by way of Eq. (6).

As can been seen from Table 4, BE-IDC achieved the smallest average threshold, $\bar {\hat {d}}=15.04$, which was about 1/5 of the fixed threshold. In addition, BE-IDC had the minimum MSPE of 1.92.

**Table 4:** Strict and individual statistical power for methods using fixed or adaptive thresholds for Example 2

Methods	Average $\hat {d}$	*P*_*a*_	*P*_100_	*P*_200_	*P*_300_	*P*_400_	*P*_500_	Average MSPE
DC-SIS (*d*=74)	74	97%	100%	99%	100%	99%	99%	3.67
IDC-SIS (*d*=74)	74	100%	100%	100%	100%	100%	100%	2.70
BE-IDC	15.04	100%	100%	100%	100%	100%	100%	1.92

### Example 3

To increase the rigor of the above two examples, we 1) weakened the signal of the influential features; and 2) increased the number of influential features. The SNPs were generated similar as in Example 2, except the correlation structure between SNPs was a little more complex, cov(*u*_*ij*_,*u*_*ik*_)=0.2^|*j*−*k*|^;*j,k*=1,…,*p*. We fix the indices of ten truly influential SNPs from *j*∈{100,200,...,1000}. The phenotype **y** and the truly influential SNPs were directly connected using a similar model as Example 1, but we took the indicator function to accommodate the categorical features, $y_{i} = \sum _{j} (\beta _{j} I(X_{ij} = 1) + 2\beta _{j} I(X_{ij} = 2)) + \epsilon $, where *ε*∼*N*(0,1). The coefficient *β*_*j*_ was randomly generated from Uniform(2,3), where the magnitudes of these coefficients were much weaker than those used in Example 1. Example 3 was simulated for *p*=2,000 SNPs, with ten influential and 1990 features as noise.

Table 5 summarizes the simulation results and the comparisons of fixed and adaptive thresholds for Example 3. If using a threshold of 37 (or 74), the DC-SIS approach only achieved a power as low as 12% (or 44%). Apparently, some feature like **X**_10_ seems to have very weak signal and trapped the DC-SIS. The results of IDC-SIS are dramatically better than those of the DC-SIS. However, the power of IDC-SIS is only 92% if using a fixed threshold of [ *n*/*logn*]=37. It indicates that IDC-SIS includes 16 more features in average than the $\bar {\hat {d}}=21.35$ estimated by BE-IDC, but was still unable to simultaneously detecting all ten influential features among all 100 replicates. To achieve a 100% power as the BE-IDC did, the IDC-SIS had to increase the threshold to 2[ *n*/*logn*]=74, but it sacrificed 53 more unnecessary noise features on average than BE-IDC.

**Table 5:** Strict and individual statistical power for methods using fixed or adaptive thresholds for Example 3

Methods	Average $\hat {d}$	*P*_*a*_	*P*_100_	*P*_200_	*P*_300_	*P*_400_	*P*_500_
DC-SIS (*d*=37)	37	12%	100%	83%	93%	100%	100%
DC-SIS (*d*=74)	74	44%	100%	99%	100%	100%	100%
IDC-SIS (*d*=37)	37	92%	100%	100%	98%	100%	100%
IDC-SIS (*d*=74)	74	100%	100%	100%	100%	100%	100%
BE-IDC	21.35	100%	100%	100%	100%	100%	100%
Methods	*P*_600_	*P*_700_	*P*_800_	*P*_900_	*P*_1000_	Average MSPE	
DC-SIS (*d*=37)	100%	81%	99%	100%	22%	4.23	
DC-SIS (*d*=74)	100%	98%	100%	100%	46%	3.68	
IDC-SIS (*d*=37)	100%	95%	99%	100%	100%	1.44	
IDC-SIS (*d*=74)	100%	100%	100%	100%	100%	1.84	
BE-IDC	100%	100%	100%	100%	100%	1.17	

### Analysis of *Arabidopsis* data

The BE-IDC procedure was applied to select the most influential SNPs for a continuous trait of the *Arabidopsis thaliana* disease-resistance phenotype, lesioning and *FRIGIDA* expression (*FRI*), with 164 inbred lines and 216,130 SNPs covering the entire genome. These data are publicly available from the link (http://arabidopsis.usc.edu). Two traditional statistical models have been implemented on this same dataset, i.e., the non-parametric Wilcoxon rank-sum test and a linear mixed model implemented in EMMA (Supplementary material of Atwell et al. [23]).

The four influential polymorphisms that were selected by BE-IDC are summarized in Table 6. Using the *Arabidopsis* Genome Initiative (AGI) genetic map and the *Arabidopsis* information resource (TAIR.org, verified on 9/5/2016) GBrowse database, we matched our significant findings with three genes. The rank 1 SNP lay approximately 217 bp away from the *FRIGIDA* region (269026 - 271503 bp). Like all other approaches working on this same dataset, the BE-IDC procedure also identified exactly the same position (Chr 4, 268809 bp) as reported by current literature. This SNP was associated with the highest peak on the Manhattan plot by all approaches, including BE-IDC. Besides the unanimous agreement on this peak, we detected three new positions that were not found by these traditional approaches for the same dataset. In particular, the rank 4 SNP lay exactly within the single large exon of *FRIGIDA* or *FLOWERING LOCUS A (FLA)* region (269026 - 271503 bp), which locates the expected gene more closely than the previously discussed highest peak. Specifically, FRI encodes a major determinant of natural variation in *Arabidopsis* flowering time and has been reported to regulate this lesioning and *FRIGIDA* expression trait under analysis. Additionally, we also found that the rank 2 and rank 3 SNPs lay within exon4 and intron2 in the neighboring *RNAHELICASE-LIKE 8 (RH8)* gene (274257 - 278737 bp), respectively.

**Table 6:** Influential SNPs selected by BE-IDC based on AGI physical map (TAIR.org)

Rank	Chr	SNP pos (bp)	Gene	Distance to gene (bp)
1	4	268809	*FRI* or *FLA*	-217
2	4	276143	*RH8*	0
3	4	275349	*RH8*	0
4	4	269260	*FRI* or *FLA*	0

**Fig. 1:**
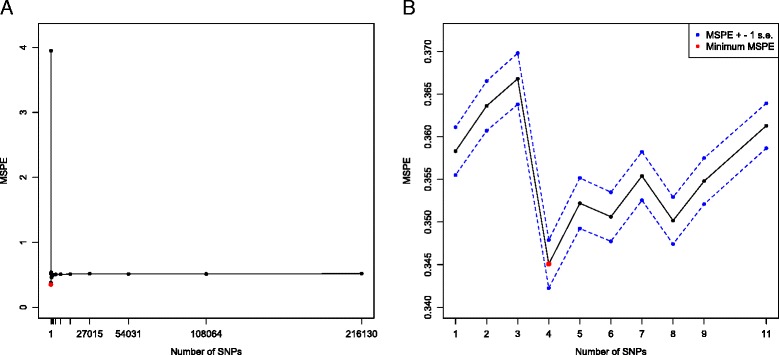
The MSPE plot. **a** The MSPE versus the number of SNPs on the interval [1, 216,130]; **b** Magnification of the MSPE over the small interval [1, 11], surrounding the minimum MSPE region. The *red point* is the final threshold determination spot (with size $\hat {d}=4$) achieving the minimum MSPE 0.34. The *black solid curve* is the traditional MSPE plot, and the *blue dash curve* is the MSPE +/- 1 standard error plot. When the model size is 103, the MSPE has the maximum value 3.95

**Fig. 2:**
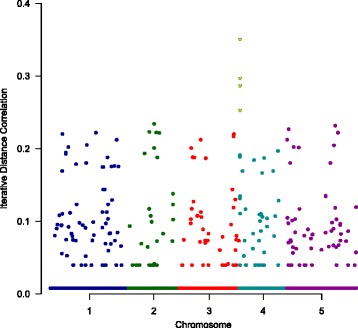
The Manhattan plot. The Manhattan plot of the FRI expression along the whole genome, based on the Dcorr measures of 216,130 SNPs against each SNP’s chromosomal position. Chromosomes are shown in alternate colors. The top four SNPs represented by the yellow triangles are finally selected by the BE-IDC procedure

Figure 1 a illustrates the MSPE plot versus the number of SNPs, covering the entire search region, during which the backward elimination removed SNPs from 216,130 to 1 iteratively at a drop rate of 50%. We observed that the minimum MSPE is achieved at a very crowded region where the number of SNPs is small. After magnifying this crowded region (Fig. 1 b), the optimal spot $\hat {d}=4$ is recognized. Figure 2 demonstrates the Manhattan plot of the continuous *FRI* trait along the entire genome, based on the iterative Dcorr values of 216,130 SNPs against their individual physical chromosomal position. Unlike the traditional Manhattan plot, we did not use the p-value and −*log*(0.05) horizontal line to determine the significance. Instead, we used the adaptive threshold determined by the BE-IDC procedure (marked in yellow triangle). Unlike the regular Manhattan plot having dense signals, Fig. 2 looks very sparse. We knew that the whole genome data had low signal-to-noise ratio and expected that the majority of the noise SNPs could be dramatically filtered out by the proposed BE-IDC approach. We also knew that IDC-SIS iteratively regressed unselected SNPs on selected SNPs and regained information from the residuals. It was also known that the residuals contain much weaker information than the original data. This explains why the majority of noise SNPs were close to 0 and why Fig. 2 looks sparse.

## Discussion

As the level of difficulty increased from Example 1 to Example 3, the average thresholds $\bar {\hat {d}}$ estimated by BE-IDC automatically became larger, causing the power to approach 100%. Comparing the different results of the three simulation examples, we confirmed that BE-IDC was indeed able to flexibly and adaptively adjust its estimated threshold value according to the specific scenarios of different datasets. On the contrary, the fixed threshold approaches failed to give a clear and flexible threshold determination. For the real data analysis, where the truth is never known, this data-driven ability is crucial. If a threshold is set too small, influential SNPs will be neglected and power will be decreased (see *d*=37 of Table 5); on the contrary, if the threshold is set too large, too much of the noise will be mistreated as influential features (i.e., false discovery) (see Tables 1 and 4).

BE-IDC works well for SNPs with either binary feature (see Additional file 1: Supplementary example 3 and Table S3) or categorical feature (see Example 2 and Table 4; Example 3 and Table 5, and Additional file 1: Supplementary example 2 and Table S2), as well as continuous features (see Example 1 and Table 1) such as age or BMI, among others [5, 34]. As for the phenotype, BE-IDC approach proposed in this article is mainly targeted for continuous phenotype/trait, hence the models, simulations, and real data all focus on continuous traits. However, we tried one simulation study with a categorical phenotype and noticed that the results were also nice (see Additional file 1: Supplementary example 1 and Table S1). We will leave this categorical trait for future examination. This article focuses on the selection of a small subset of influential genes that still achieve sufficient prediction accuracy for new observations, which is a common interest in plant breeding in crop, plant, and cattle species or disease diagnosis and prevention in clinical practices [4, 35–39]. For the case of several hundreds of influential SNPs all individually having small effect, the proposed BE-IDC may not be feasible and we will consider it in future work.

## Conclusion

This article proposes a BE-IDC procedure with the aims of (1) selecting the smallest possible set of influential genes from a big pool that are not only associated with the analyzed phenotype, but also enable accurate prediction for new observations; and (2) determining an adaptive threshold effectively separating influential SNPs from the noise SNPs. An approach with accurate prediction capability will make the results obtained in one dataset be reproduced better in a different dataset. The difficulties that BE-IDC overcomes are as follows: issues of ultrahigh dimensionality when the number of SNPs is in the tens of thousands or even in the millions, but the number of observations is in the hundreds or the thousands; detecting signals from a sparse structure (i.e., signal-to-noise ratio is very weak); and detecting truly important SNPs that are confounded by noise due to strong linkage disequilibrium.

## Additional file

Additional file 1 Supplementary examples. (PDF 122 kb)

## Abbreviations

BE-IDC: Backward elimination iterative distance correlation
Dcorr: Distance correlation
DC-SIS for distance correlation based sure independence feature screening; FRI: *FRIGIDA* expression
IDC-SIS: Iterative distance correlation feature screening approach
LD: Linkage disequilibrium
MSPE: Mean square prediction error
OOB: Out of bag observations
RH8: *RNAHELICASE-LIKE 8*
SCAD: Smoothly clipped absolute deviation
SNP stands: single nucleotide polymorphisms
