# Supplementary material for: An adaptive threshold determination method of feature screening for genomic selection
Source: BMC Bioinformatics. 2017 Apr 12;18:212. doi: 10.1186/s12859-017-1617-9 (PMC5389084; doi:10.1186/s12859-017-1617-9)
Supplement: Additional file 1 — Supplementary examples. (PDF 122 kb) [file 12859_2017_1617_MOESM1_ESM.pdf]

Additional file 1

Supplementary example 1

To assess the performance of BE-IDC on a categorical phenotype, and also make the comparison fair, we used exactly the same model as Zhong et al. [27]. The categorical phenotype y was generated from the model

$$y = \begin{cases} 1, & \text{if} \quad Y^* \in (-\infty, -3); \\ 2, & \text{if} \quad Y^* \in [-3, 0); \\ 3, & \text{if} \quad Y^* \in [0, 3); \\ 4, & \text{if} \quad Y^* \in [3, +\infty), \end{cases} \tag{S1}$$

where the continuous phenotype Y^* and all features are still generated following exactly the same rule as Example 1, i.e., $Y^* = 5X_1+5X_2+5X_3-15\sqrt{\rho}X_4+\epsilon$. Similar to Example 1, the first four features are influential ones and all other 4,996 features are noise. As observed from Table S1, the results of Supplementary example 1 were very similar to those of Example 1. The adaptive thresholds achieved the same power as the fixed thresholds, but used much fewer features. It is encouraging to see that the BE-IDC still achieved 100% power for a categorical phenotype. However, BE-IDC now involved more features ($\bar{\hat{d}} = 23.3$), compared to that of Example 1 ($\bar{\hat{d}} = 5.54$). Since the categorical phenotype is connected with the feature through an indirect intermediate model (S1) (i.e., the association structure of Supplementary example 1 is more rigorous than that of Example 1), it is expected that the threshold of Supplementary example 1 is larger than that of Example 1. However the fixed threshold approach failed to adaptively change the threshold according to different scenarios. We did not compute the MSPE for this example because its trait is categorical.

Table S1 Strict and individual statistical power for methods using fixed or adaptive thresholds for Supplementary example 1.

Methods	Average $\hat{d}$	$\mathbf{P_a}$	$\mathbf{P_1}$	$\mathbf{P_2}$	$\mathbf{P_3}$	$\mathbf{P_4}$
DC-SIS $(d = 74)$	74	0%	100%	100%	99%	0%
IDC-SIS $(d = 74)$	74	100%	100%	100%	100%	100%
BE-IDC	23.30	100%	100%	100%	100%	100%

Supplementary example 2

In this example, we simulated the SNPs whose genotypes are satisfied by the Hardy-Weinberg Equilibrium (HWE) with the minor allele frequency randomly selected from Uniform(0.3,0.5). All the other parts of this example were the same as Example 2. In Table S2 the performance of BE-IDC is stable compared to the results of Example 2.

Table S2 Strict and individual statistical power for methods using fixed or adaptive thresholds for Supplementary example 2.

Methods	Average $\hat{d}$	$\mathbf{P_a}$	$\mathbf{P_1}$	$\mathbf{P_2}$	$\mathbf{P_3}$	$\mathbf{P_4}$	$\mathbf{P_5}$	Average MSPE
DC-SIS $(d = 74)$	74	100%	100%	100%	100%	100%	100%	2.24
IDC-SIS $(d = 74)$	74	100%	100%	100%	100%	100%	100%	2.50
BE-IDC	15.39	100%	100%	100%	100%	100%	100%	1.87

Supplementary example 3

To perfectly mimic the Arabidopsis data or similar situations when the heterozygote is not showing up, we simulated 2,000 SNPs based on the Bernoulli distribution with minor allele frequency randomly selected from Uniform(0.3, 0.5). The phenotype y and ten truly influential SNPs were directly connected by

$$y_i = \sum_j \beta_j I(X_{ij} = 1) + \epsilon,$$

where $\epsilon \sim N(0, 1)$, $j \in \{100, 200, ..., 1000\}$, and $\beta_j \sim$ Uniform$(2, 3)$. Table S3 summarizes the simulation results and the comparisons of fixed and adaptive thresholds for Supplementary example 3. BE-IDC still achieves the highest power using the smallest model size for the SNPs generated under Bernoulli distribution.

Table S3 Strict and individual statistical power for methods using fixed or adaptive thresholds for Supplementary example 3.

Methods	Average $\hat{d}$	$\mathbf{P_a}$	$\mathbf{P_{100}}$	$\mathbf{P_{200}}$	$\mathbf{P_{300}}$	$\mathbf{P_{400}}$	$\mathbf{P_{500}}$
DC-SIS ($d = 37$)	37	52%	99%	99%	100%	100%	100%
DC-SIS ($d = 74$)	74	73%	100%	100%	100%	100%	100%
IDC-SIS ($d = 37$)	37	89%	99%	100%	99%	100%	100%
IDC-SIS ($d = 74$)	74	97%	100%	100%	100%	100%	100%
BE-IDC	21.93	97%	100%	100%	100%	100%	100%
Methods	$\mathbf{P_{600}}$	$\mathbf{P_{700}}$	$\mathbf{P_{800}}$	$\mathbf{P_{900}}$	$\mathbf{P_{1000}}$	Average MSPE	
DC-SIS ($d = 37$)	100%	55%	100%	99%	100%	1.84	
DC-SIS ($d = 74$)	100%	73%	100%	100%	100%	2.18	
IDC-SIS ($d = 37$)	100%	95%	100%	96%	100%	1.40	
IDC-SIS ($d = 74$)	100%	99%	100%	98%	100%	1.84	
BE-IDC	100%	97%	100%	99%	100%	1.19	
